# Polycythemia Vera, Thrombophilia, CTEPH, Cerebral Venous Sinus Thrombosis and Vertebral Artery Occlusion: A Case-Illustrated Narrative Review of Competing Thrombotic and Hemorrhagic Risks

**DOI:** 10.3390/life16071149

**Published:** 2026-07-11

**Authors:** Razvan-Adrian Bertici, Amalia Ridichie, Nicoleta Sorina Bertici, Dragos Catalin Jianu, Georgiana Munteanu, Traian Flavius Dan, Adelina Miron, Lavinia Mihnea, Nicoleta Iacob, Ovidiu Fira-Mladinescu

**Affiliations:** 1 Doctoral School, “Victor Babes” University of Medicine and Pharmacy Timisoara, Eftimie Murgu Square No. 2, 300041 Timisoara, Romania; razvan.bertici@umft.ro; 2 Center for Research and Innovation in Personalized Medicine of Respiratory Diseases, Department of Infectious Diseases, “Victor Babes” University of Medicine and Pharmacy Timisoara, Eftimie Murgu Square No. 2, 300041 Timisoara, Romania; mladinescu@umft.ro; 3 Centre for Cognitive Research in Neuropsychiatric Pathology, Department of Neurosciences, “Victor Babes” University of Medicine and Pharmacy Timisoara, Eftimie Murgu Square No. 2, 300041 Timisoara, Romania; munteanu.georgiana@umft.ro (G.M.); traian.dan@umft.ro (T.F.D.); 4 First Department of Neurology, “Pius Brînzeu” Clinical Emergency County Hospital, Liviu Rebreanu Avenue No. 156, 300736 Timisoara, Romania; adelina.miron@rezident.umft.ro (A.M.); lavinia.mihnea@rezident.umft.ro (L.M.); 5 Analytical Chemistry Division, Faculty of Pharmacy, “Victor Babes” University of Medicine and Pharmacy Timisoara, Eftimie Murgu Square No. 2, 300041 Timisoara, Romania; amalia.ridichie@umft.ro; 6 Advanced Instrumental Screening Center, Faculty of Pharmacy, “Victor Babes” University of Medicine and Pharmacy Timisoara, Eftimie Murgu Square No. 2, 300041 Timisoara, Romania; 7 Pulmonology Division, Department of Infectious Diseases, “Victor Babes” University of Medicine and Pharmacy Timisoara, Eftimie Murgu Square No. 2, 300041 Timisoara, Romania; 8 Second Department of Pulmonology, Clinical Hospital of Infectious Diseases and Pulmonology “Victor Babes” Timisoara, Gheorghe Adam Street No. 13, 300310 Timisoara, Romania; 9 First Division of Neurology, Department of Neurosciences, “Victor Babes” University of Medicine and Pharmacy Timisoara, Eftimie Murgu Square No. 2, 300041 Timisoara, Romania; 10 Department of Multidetector Computed Tomography and Magnetic Resonance Imaging, Scanexpert, 300627 Timisoara, Romania; nicoiacob@yahoo.co.uk

**Keywords:** polycythemia vera, thrombophilia, chronic thromboembolic pulmonary hypertension, CTEPH, chronic hypoxia, cerebral venous sinus thrombosis, CVST, vertebral artery occlusion

## Abstract

Background: The coexistence of systemic prothrombotic disorders, chronic thromboembolic pulmonary hypertension (CTEPH), chronic hypoxia, and cerebrovascular thrombosis creates complex diagnostic and therapeutic challenges. Case summary: We report the case of a 52-year-old woman with JAK2V617F-positive polycythemia vera, inherited thrombophilic abnormalities, recurrent pulmonary thromboembolism progressing to severe CTEPH, chronic hypoxemia, cerebral venous sinus thrombosis, and right vertebral artery occlusion. Management challenge: The case illustrates persistent thrombotic risk despite anticoagulation, the need for disease-directed cytoreduction, limited access to CTEPH-directed interventional treatment, neurological vulnerability despite preserved brain parenchymal integrity, and the narrow therapeutic margin created by the combined use of anticoagulant, cytoreductive, and pulmonary vasodilator therapy. Particular emphasis is placed on the competing risks of recurrent thrombosis and hemorrhagic complications, especially in the cerebrovascular territory. Conclusion: This case highlights the need for repeated multidisciplinary reassessment in patients with overlapping hematological, pulmonary, and neurological vascular disease. Improved survival in patients with severe multisystemic conditions may increase the clinical relevance of complex presentations requiring coordinated management. Further evidence is needed to support safer, more standardized treatment strategies for patients requiring simultaneous control of thrombosis, pulmonary vascular disease, myeloproliferation, hypoxia, and treatment-related bleeding risk.

## 1. Introduction

Prothrombotic risk factors, whether inherited or acquired, may act independently or, more importantly, synergistically to produce a hypercoagulable state. This predisposes patients to arterial and venous thrombosis across multiple vascular territories, including deep vein thrombosis, pulmonary embolism, arterial ischemic stroke, cerebral venous thrombosis, and thrombosis in other atypical locations. When several prothrombotic mechanisms coexist, the clinical presentation may become complex, recurrent, and multisystemic [1,2,3].

Polycythemia vera is a chronic myeloproliferative neoplasm associated with an increased risk of both arterial and venous thrombosis. This thrombotic tendency is multifactorial. Increased red cell mass contributes to elevated blood viscosity, reduced blood flow, and enhanced interaction between platelets, leukocytes, and the vascular endothelium. In addition, platelet activation, leukocytosis, endothelial dysfunction, inflammation, and Janus kinase 2 (JAK2)—driven myeloproliferation may further amplify the prothrombotic state [4,5,6,7,8].

Inherited thrombophilias represent another important contributor to thrombotic risk by altering physiological anticoagulant pathways, coagulation activation, or fibrinolytic balance. Clinically relevant inherited thrombophilias include deficiencies of natural anticoagulants such as protein C, protein S, and antithrombin, as well as selected coagulation factor variants. Other genetic polymorphisms, including Factor V and methylenetetrahydrofolate reductase (MTHFR), may contribute to an individual’s prothrombotic background, although their independent clinical significance is currently debated and should be interpreted in the context of the overall risk profile [9,10,11,12].

The coexistence of polycythemia vera with inherited thrombophilia can generate a severe systemic prothrombotic state, particularly in patients with recurrent thrombotic events or thrombosis involving multiple vascular beds. This overlap creates a major therapeutic challenge, especially when long-term anticoagulation is required but bleeding risk, thrombocytopenia, or treatment-related adverse effects limit its safe use [13].

Large pulmonary emboli or recurrent smaller thromboembolic events may lead, over time, to chronic obstruction and remodeling of the pulmonary vascular bed, resulting in chronic thromboembolic pulmonary hypertension (CTEPH). CTEPH is classified as a precapillary form of pulmonary hypertension and represents a severe, potentially progressive complication of unresolved pulmonary thromboembolism. Its pathophysiology involves the persistence of organized thrombotic material within the pulmonary arteries, accompanied by secondary microvascular remodeling and increased pulmonary vascular resistance, ultimately leading to progressive right ventricular pressure overload and dysfunction [14,15].

Although pulmonary endarterectomy represents the main potentially curative treatment for CTEPH, its indication depends on the anatomical accessibility of thrombotic lesions, surgical risk, comorbidities, and the overall clinical profile of the patient. Balloon pulmonary angioplasty may also represent an important interventional option in selected cases, particularly in patients with inoperable disease or residual pulmonary hypertension after surgery. However, in patients with a severe systemic prothrombotic state, multiple comorbidities, and high therapeutic complexity, pulmonary vasodilator therapy may represent the safest and most feasible therapeutic approach for long-term stabilization, especially when invasive procedures carry increased risk or are not considered suitable. In this context, medical therapy aims to reduce pulmonary vascular resistance, improve functional status, and limit the consequences of persistent pulmonary hypertension, while anticoagulation remains essential to prevent recurrent thromboembolic events [16,17,18].

The chronic impairment of pulmonary circulation in CTEPH may also lead to persistent hypoxemia, frequently requiring long-term oxygen therapy. Chronic hypoxia has important systemic consequences and may increase neurological vulnerability over time. At the cerebral level, sustained oxygen deprivation may contribute to hypoxic encephalopathy, cognitive decline, impaired attention, reduced processing speed, fatigue, and overall functional deterioration. Therefore, in patients with CTEPH and severe prothrombotic disease, neurological symptoms must be interpreted not only in relation to focal arterial or venous cerebrovascular events, but also in the broader context of chronic hypoxia and its potential cognitive impact [19,20].

Cerebral venous and sinus thrombosis (CVST) represents a rare form of stroke with highly variable clinical presentation, depending on the venous territory involved, the degree of impaired venous drainage and the presence of parenchymal lesions. The most frequent clinical presentation is related to intracranial hypertension, usually manifested by headache, nausea, vomiting, papilledema, or visual symptoms. Other important clinical syndromes include focal neurological deficits, seizures, encephalopathy, and, in selected cases, cavernous sinus syndrome. In patients with chronic hypoxia, the interpretation of neurological symptoms may be particularly difficult, as altered mental status, fatigue, cognitive slowing, or encephalopathic features may result from cerebral venous congestion, hypoxic mechanisms, or a combination of both [21,22,23].

Vertebral artery occlusion may result from several mechanisms, including atherosclerosis, dissection, embolism, or thrombosis in the context of systemic hypercoagulability. Depending on the affected segment and collateral circulation, vertebral artery occlusion may compromise posterior circulation flow and present with vertigo, dizziness, ataxia, nausea, vomiting, visual disturbances, or other brainstem and cerebellar signs [24,25].

The coexistence of venous and arterial cerebrovascular lesions is particularly concerning, as in such patients, the risk of cerebral ischemia, venous infarction, hemorrhagic transformation, or progressive neurological deterioration is significant and may be further amplified by chronic hypoxia, impaired vascular reserve, and the difficulty of maintaining a safe but effective antithrombotic strategy [26].

At the same time, the combined treatment required for these overlapping hematological, pulmonary, and neurological conditions may expose the patient to significant hemorrhagic risk. This risk is especially important in the cerebrovascular territory, where anticoagulation, thrombocytopenia, impaired platelet function, and pre-existing vascular lesions may favor intracranial bleeding or hemorrhagic transformation.

The aim of this article is to review the available literature regarding the complex association between systemic prothrombotic disorders, chronic thromboembolic pulmonary hypertension, chronic hypoxia, and cerebrovascular thrombotic complications, with particular emphasis on therapeutic decision-making when thrombotic and hemorrhagic risks coexist. In parallel, we present an ongoing clinical case that illustrates the difficulty of multidisciplinary management of such a patient.

## 2. Review Strategy

Data regarding polycythemia vera and its secondary arterial and venous thrombotic complications are well established in the current literature. The thrombotic risk in polycythemia vera is multifactorial and may be further amplified by the presence of additional inherited thrombophilic factors [1,4,10]. In such patients, acquired and inherited prothrombotic mechanisms may overlap, increasing not only the overall thrombotic burden, but also the probability of recurrent, atypical, or multisystemic vascular events [2,3,13].

However, the association of all the pathologies discussed in the present article remains extremely rare. Polycythemia vera itself is an uncommon disease, with an estimated incidence of 0.68 to 2.6 per 100,000 per year and a prevalence ranging from 0.49 to 46.88 per 100,000 individuals [27,28,29]. Cerebral venous sinus thrombosis is also rare, with an estimated incidence of approximately 0.2 to 2 cases per 100,000 individuals per year [30], while chronic thromboembolic pulmonary hypertension has been reported to occur in approximately 2.7% of survivors after pulmonary embolism [31]. Considering the rarity of each individual pathology, their coexistence in the same patient represents an exceptional clinical scenario, particularly when associated with inherited thrombophilia and arterial cerebrovascular occlusion.

A narrative literature search was performed in PubMed, Scopus, and Web of Science. The search included publications from January 2016 to early June 2026, corresponding to the preceding 10-year period. The search strategy included combinations of the following terms: “polycythemia vera”, “thrombophilia”, “chronic thromboembolic pulmonary hypertension”, “CTEPH”, “cerebral venous sinus thrombosis”, “CVST”, “arterial occlusion”, and “vertebral artery occlusion”. Articles, reviews, and case reports were considered eligible, while retracted publications were excluded. Only English-language publications were considered. Because the present article was designed as a narrative review integrated with an illustrative clinical case, rather than as a systematic review or meta-analysis, a formal PRISMA-based selection process was not applied. Similarly, a PICO framework was not used because the objective was not to answer a narrowly defined interventional or comparative clinical question, but to contextualize a rare multisystemic association and discuss the therapeutic challenges generated by overlapping prothrombotic, pulmonary vascular, neurological, and hemorrhagic mechanisms. Within the limits of this narrative search, we did not identify reports describing the full association of these pathologies in a single patient, nor articles specifically discussing the multidisciplinary management difficulties generated by the coexistence of high thrombotic risk and treatment-related hemorrhagic risk in this context.

The next step was to evaluate the available literature regarding clinical management strategies for highly prothrombotic states, as well as the therapeutic options available for each associated pathology. Particular attention was given to treatment-related adverse effects, especially hemorrhagic risk, possible drug interactions, and the challenges of chronic management in patients with multiple overlapping diseases. In such cases, therapeutic decisions are made within a narrow safety margin, as treatments required for one pathology may worsen the risk profile of another. This creates a complex clinical scenario in which anticoagulation, cytoreductive therapy, pulmonary vasodilator treatment, platelet monitoring, bleeding risk, and neurological protection must be balanced simultaneously.

## 3. Case Presentation

### 3.1. Patient Background

We present the case of a 52-year-old woman from a rural area, a non-smoker with class I obesity and a complex prothrombotic profile, who developed recurrent venous and arterial thrombotic and thromboembolic events across multiple vascular territories.

The earliest relevant elements in the patient’s medical history were two spontaneous miscarriages of unknown etiology, which were not further investigated at the time. Subsequently, around 2000, she was diagnosed with chronic venous insufficiency and deep vein thrombosis, for which oral anticoagulant therapy with acenocoumarol was prescribed for three months. Together, these events were later regarded as early clinical indicators of an underlying prothrombotic predisposition. Her medical history was also notable for arterial hypertension.

Family history revealed obesity and chronic venous insufficiency in both her mother and sister. No other relevant family medical history was reported.

### 3.2. Acute Pulmonary Embolism

Since 2014, the patient had experienced recurrent episodes of dyspnea, dry cough, and asthenia, which were managed exclusively by her general practitioner, without further diagnostic investigations.

**Figure 1:**
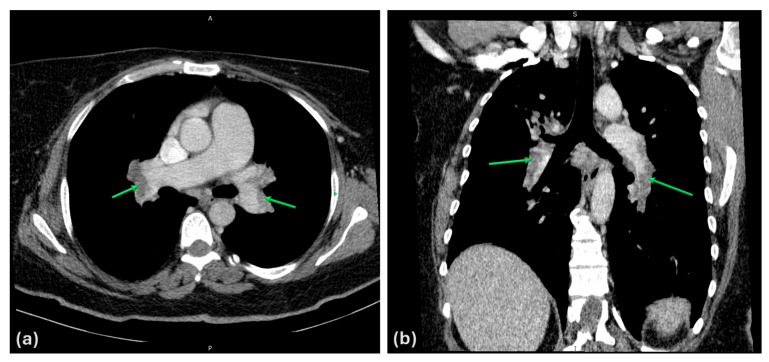
Chest CT angiography performed in 2017 shows acute massive pulmonary embolism (arrows) in the bilateral pulmonary arteries on axial (**a**) and coronal reconstructions (**b**).

The first clearly documented thromboembolic event occurred in 2017, when the patient presented to the emergency department with sudden-onset severe dyspnea and presyncope. Chest computed tomography (CT) angiography confirmed massive acute pulmonary thromboembolism involving the bilateral pulmonary arteries (Figure 1).

Following acute management of this episode, long-term oral anticoagulant therapy with acenocoumarol was initiated. Treatment continued until 2019, when acenocoumarol was discontinued because of gastrointestinal intolerance and replaced with direct oral anticoagulant therapy with apixaban. The intolerance consisted of gastrointestinal symptoms such as abdominal pain and diarrhea, without gastrointestinal bleeding or other hemorrhagic complications during acenocoumarol treatment.

Over the subsequent years, the patient experienced recurrent respiratory symptoms, although imaging studies revealed no new acute thromboembolic events and showed only persistent chronic thrombotic changes. No emergency department presentations were recorded during this period. Despite the absence of acute radiological findings, her symptoms progressively worsened, with dyspnea occurring even during mild exertion.

### 3.3. Chronic Thromboembolic Pulmonary Hypertension

In 2024, the patient was referred by her general practitioner for further cardiopulmonary evaluation because of progressive respiratory deterioration and worsening functional capacity.

Transthoracic echocardiography showed elevated pulmonary artery pressures and right-sided chamber dilatation, with an estimated systolic pulmonary artery pressure of 60 mmHg.

**Figure 2:**
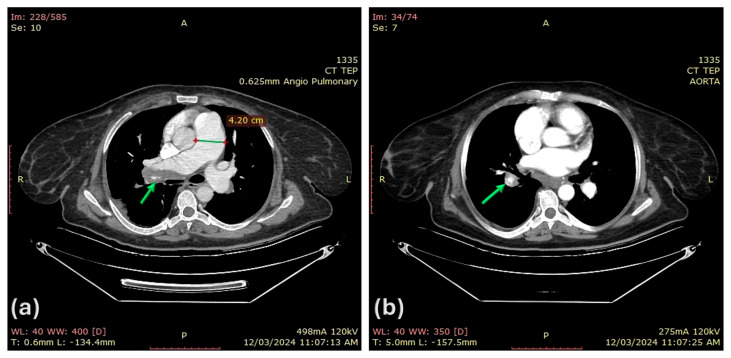
The chest CT angiography from 2024 reveals an enlarged pulmonary artery trunk and thrombosis (arrows) in the right pulmonary artery (**a**) and lower lobar pulmonary artery (**b**).

Subsequent right-heart catheterization confirmed severe precapillary pulmonary hypertension, with a mean pulmonary arterial pressure (mPAP) of 57 mmHg, systolic pulmonary arterial pressure (sPAP) of 90 mmHg, pulmonary arterial wedge pressure (PAWP) of 14 mmHg, and pulmonary vascular resistance (PVR) of 14 Wood units. The patient was then referred to the regional pulmonary arterial hypertension center, where chronic thromboembolic pulmonary hypertension, classified as Group 4 pulmonary hypertension, was diagnosed. Repeated chest CT angiography further supported this diagnosis, demonstrating enlargement of the pulmonary artery trunk (42 mm) and persistent thrombotic material within the right pulmonary artery and lower lobar pulmonary arterial branches (Figure 2).

Given the lack of local availability of pulmonary endarterectomy or balloon pulmonary angioplasty, as well as the patient’s refusal to be transferred to an international expert center, management was limited to targeted vasodilator therapy and supportive care. Accordingly, riociguat was initiated, and supplemental oxygen therapy was prescribed for home use as needed.

**Figure 3:**
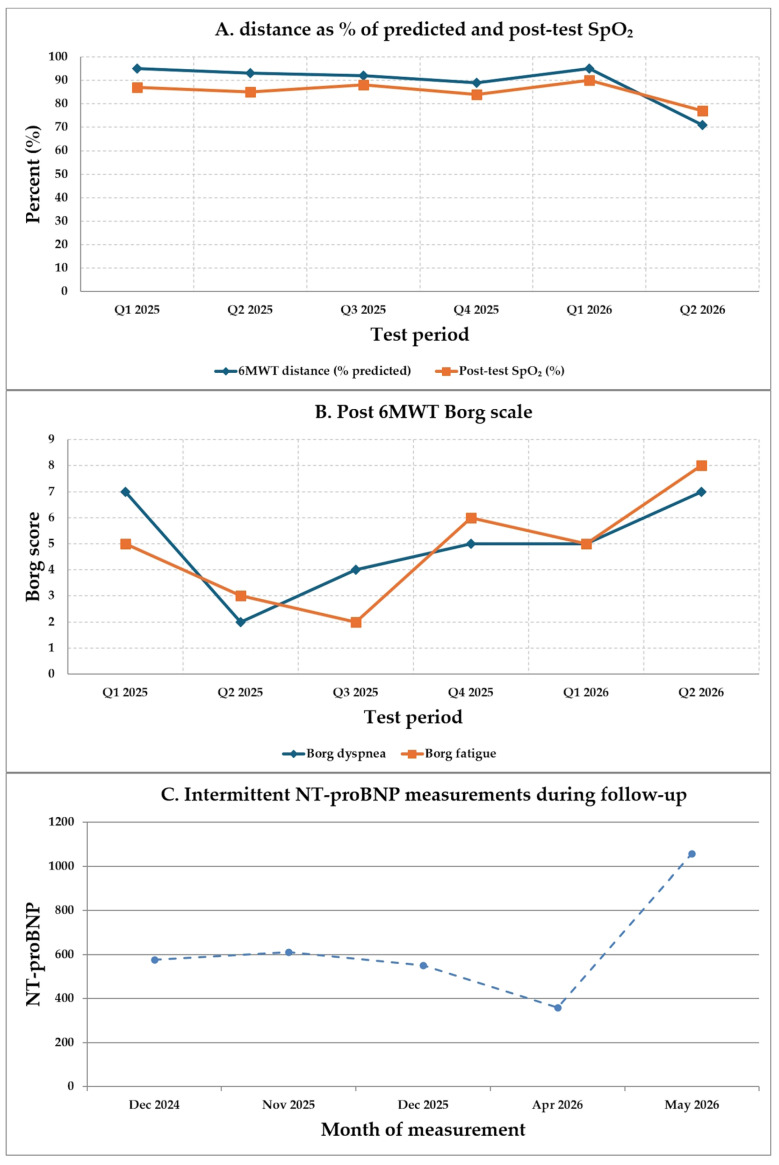
Functional and cardiopulmonary follow-up during dual pulmonary vasodilator therapy: (**A**) Serial quarterly 6-min walk test (6MWT) assessments showing distance as percentage of predicted value and post-test peripheral oxygen saturation. (**B**) Post-6MWT Borg dyspnea and fatigue scores. (**C**) Intermittent NT-proBNP measurements obtained during cardiopulmonary follow-up. NT-proBNP values were interpreted together with functional performance, oxygenation, imaging findings, and hematological status.

Despite riociguat therapy, the pulmonary response remained suboptimal, with persistence of a high-risk clinical profile characterized by a 6-min walk distance below 300 m, NYHA functional class III, and elevated NT-proBNP levels above 1000 pg/mL. These findings prompted consideration of escalation to dual therapy. Consequently, at the end of 2024, continuous subcutaneous treprostinil infusion was added. Subsequent follow-up demonstrated improvement in functional status, with NYHA class improving to II/III, and serial 6-min walk test performance is presented in Figure 3.

Treatment-related adverse effects were recorded during this period, including rare, episodic, nonspecific headaches considered secondary to vasodilator therapy, associated with dizziness. However, these adverse effects did not significantly affect the patient’s overall clinical status.

### 3.4. Hematological Workup

During the same period, detailed laboratory screening revealed significant and persistent erythrocytosis, despite clinical improvement in desaturation episodes, prompting further hematological evaluation.

Bone marrow biopsy demonstrated erythro-megakaryocytic hyperplasia, and molecular testing confirmed the presence of the JAK2 V617F mutation, establishing the diagnosis of polycythemia vera. Beta-thalassemia minor was identified, and significant splenomegaly was also observed after imaging with dimensions of 175 × 182 × 110 mm.

Following hematological assessment, hydroxycarbamide was initiated to improve control of the symptomatic polycythemia vera-related prothrombotic profile, which persisted despite anticoagulant therapy.

Thrombophilia testing revealed reduced protein C activity, heterozygous Factor V H1299R and MTHFR C677T mutations, heterozygous PAI-1 4G/5G polymorphism, and the presence of the EPCR A1/A2 haplotype.

**Figure 4:**
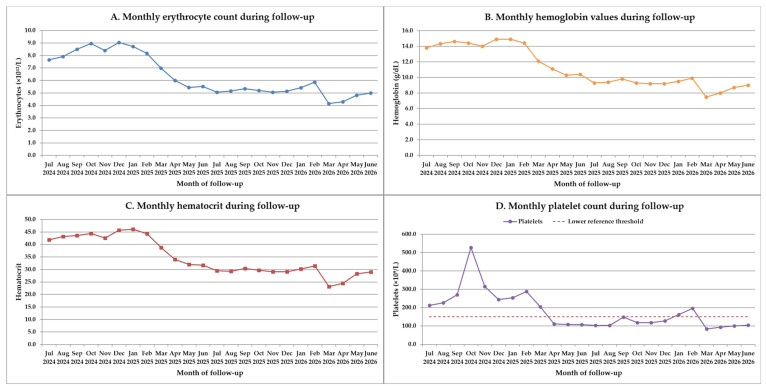
Longitudinal hematological follow-up: (**A**) Monthly erythrocyte count during follow-up. (**B**) Monthly hemoglobin values during follow-up. (**C**) Monthly hematocrit values during follow-up. (**D**) Monthly platelet count during follow-up, with the dashed line indicating the lower reference threshold.

Subsequent follow-up in 2024 showed insufficient response to hydroxycarbamide, and the decision was made to switch to ruxolitinib in combination with apixaban, given the patient’s marked prothrombotic risk, persistent myeloproliferative disease activity, and associated splenomegaly. Ruxolitinib was selected because of its targeted inhibition of the JAK/STAT pathway, with potential benefit for hematologic control, symptom burden, and splenomegaly in patients with polycythemia vera who show inadequate response or intolerance to hydroxycarbamide. Phlebotomy was not pursued after ruxolitinib achieved hematocrit control below the recommended target of 45%, with observed hematocrit and hemoglobin values described in Figure 4 during follow-up. In this context, additional phlebotomy was not expected to provide further thrombotic-risk benefit and could have worsened the patient’s limited oxygen-carrying reserve, particularly given severe CTEPH, chronic hypoxemia, beta-thalassemia minor, and the later development of clinically significant anemia.

### 3.5. Neurological Presentation

During the first quarter of 2025, the patient developed recurrent episodes of macroscopic hematuria, most likely related to thrombocytopenia. Before medical reassessment could be performed, she discontinued apixaban on her own initiative. Within the following days, she developed new neurological symptoms, including increasingly frequent headaches, vertigo with impaired balance, and transient visual disturbances. Given the clinical suspicion of transient ischemic events in the vertebrobasilar territory, an urgent neurological consultation was requested.

Neurological examination showed a conscious, cooperative patient, with a Glasgow Coma Scale score of 15, preserved spatial and temporal orientation, and no signs of meningeal irritation. The headaches were diffuse, nonspecific, recurrent, and of low-to-moderate intensity, without associated nausea or vomiting, and could not be classified as a primary headache disorder. Given the clinical context, they were considered secondary, possibly related either to vasodilator therapy, as similar episodes had been previously reported, or to a newly developed neurovascular cause. Vertigo was persistent and clinically consistent with central origin. No horizontal or vertical nystagmus, abnormal ocular movements, diplopia, ataxia, gait disturbance, or other cerebellar signs were observed at examination. No focal motor or sensory deficits, no involuntary movements and no cranial nerve abnormalities were present. No clinical visual field impairment was identified, and ophthalmological assessment confirmed the absence of persistent visual field deficit or papilledema. Associated ophthalmological findings were suggestive of Sicca syndrome, for which local symptomatic treatment was recommended.

**Figure 5:**
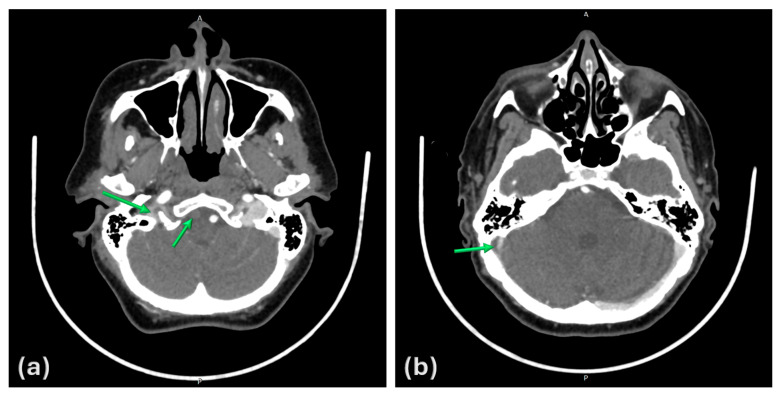
CT angiography shows thrombosis (arrows) in the right sigmoid sinus and occlusion in the V4 segment of the right vertebral artery (**a**) and thrombosis of the right transverse sinus (**b**).

**Figure 6:**
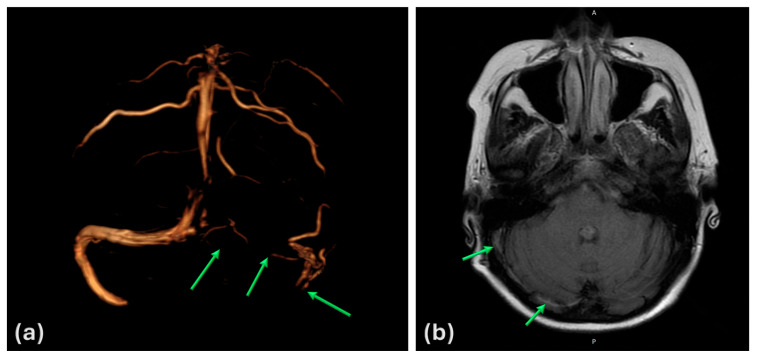
The brain MRI performed the following day revealed thrombosis in the right transverse and sigmoid sinuses on the venous angiography sequence 2D TOF ((**a**), arrows) and reduced flow in these sinuses on the FLAIR sequence ((**b**), arrows).

Emergency neuroimaging with CT angiography revealed concomitant occlusion of the right vertebral artery at the V4 segment and thrombosis of the right transverse and sigmoid sinuses. The anterior circulation remained patent, collateral circulation was preserved, and the basilar artery was not involved. No arterial ischemic lesion, venous infarction, significant cerebral edema, or hemorrhagic transformation was identified. These findings were confirmed the following day by brain magnetic resonance imaging (MRI) with MR angiographic and venographic sequences, which demonstrated persistent right vertebral artery occlusion and thrombosis of the right transverse–sigmoid sinus system (Figure 5 and Figure 6).

Anticoagulant therapy with apixaban was immediately resumed because continued anticoagulation was considered mandatory despite the recent hemorrhagic adverse event, given the substantial risk of major thrombotic complications following treatment interruption. At that time, macroscopic hematuria had subsided. A urological evaluation was performed to exclude a structural urinary tract cause, but no clinically relevant abnormality was identified. Therefore, anticoagulation was restarted under close monitoring, with approximately monthly complete blood count assessment and surveillance for recurrent hematuria or other bleeding manifestations. Based on the same risk–benefit considerations, antiplatelet therapy was not initiated. Because no arterial infarction was present and the patient already required mandatory anticoagulation, the addition of antiplatelet therapy was considered to offer uncertain incremental benefit while substantially increasing hemorrhagic risk in the context of recent macroscopic hematuria, thrombocytopenia, and ongoing bleeding vulnerability. Symptomatic treatment for vertigo was initiated, with good clinical response. Since neuroimaging showed no arterial infarction, venous infarction, significant cerebral edema, or hemorrhagic transformation, no cerebral anti-edema or depletive therapy was deemed necessary at that time.

Cognitive status was also evaluated using standardized Romanian versions of the mini-mental state examination (MMSE) and Montreal cognitive assessment (MoCA) scales in order to assess the potential impact of combined effects of chronic hypoxia and neurovascular pathology. The MMSE score was 29/30, with the lost point in the recall domain. The MoCA score was 25/30, at the limit for mild cognitive impairment, with deficits mainly in memory and attention domains.

### 3.6. Multidisciplinary Follow-Up and Therapeutic Balance

From this point onward, the central therapeutic challenge became balancing the need for continuous anticoagulation against the risk of hemorrhagic complications. The patient’s treatment involved several potentially interacting therapeutic factors, including anticoagulant therapy, cytoreductive treatment, and prostanoid pulmonary vasodilator therapy. Therefore, a compound medication-related contribution to thrombocytopenia and bleeding risk was likely.

Monthly hematological monitoring was recommended (see Figure 4), with particular attention to erythrocyte count, hemoglobin levels, and platelet count, alongside regular follow-up visits for CTEPH assessment, treatment adjustment, and neurological reevaluation at six months.

**Figure 7:**
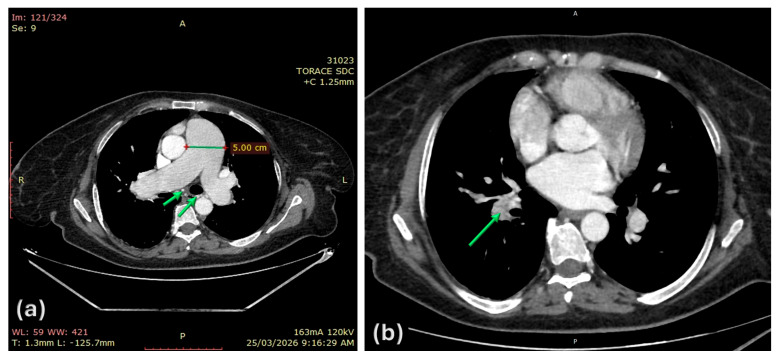
The chest CT angiography performed in 2026 shows progressive dimensional increase in the caliber of the pulmonary artery trunk (**a**) and almost complete chronic thrombosis of the left inferior lobar pulmonary artery (**b**), with MAPCA-type aorto-pulmonary arterial collateral circulation (arrows in (**a**)).

During follow-up CTEPH assessments, imaging showed persistent chronic thrombotic disease without newly developed significant pulmonary thromboembolic events. Resting oxygen saturation remained generally above 90% under pulmonary vasodilator therapy and intermittent supplemental oxygen. Nevertheless, imaging demonstrated clear disease progression, with progressive dilatation of the pulmonary artery trunk, now measuring 50 mm, associated with significant major aortopulmonary collateral arteries (MAPCA)-type aortopulmonary collateral circulation (Figure 7).

**Figure 8:**
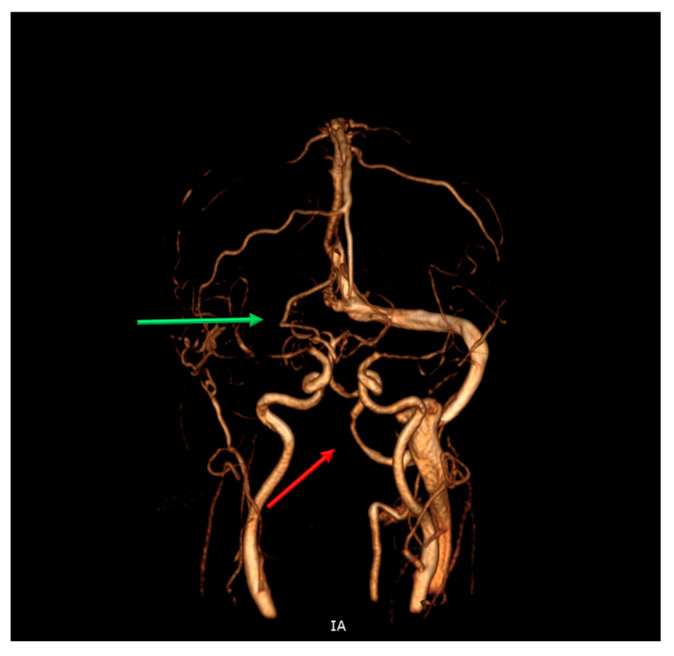
Venous MRI angiography (PHASE CONTRAST) performed after 6 months shows lack of flow in the right vertebral artery (red arrow) and in the right transverse and sigmoid sinuses (green arrow).

Neurological reassessment at 6 months demonstrated persistent chronic right vertebral artery occlusion and chronic right transverse and sigmoid sinuses thrombosis, without newly appearing imaging lesions (Figure 8).

Despite these persistent vascular findings, the clinical neurological profile improved significantly, with only rare headaches and no recurrence of central vertigo or transient visual symptoms after the initial neurological episode.

Cognitive evaluation showed results similar to the initial testing, with an MMSE score of 28/30 and a MoCA score of 25/30, with deficits affecting the same domains as previously described.

Given the recurrent and multisystemic thrombotic phenotype, the follow-up workup also included evaluation for additional acquired prothrombotic conditions. Extended antinuclear antibody IgG testing did not support an underlying systemic autoimmune or vasculitic disorder, and antiphospholipid syndrome testing was negative. Available imaging and laboratory evaluation did not reveal findings suggestive of an active malignant process.

At a recent follow-up evaluation, the patient’s clinical profile deteriorated in the context of significant anemia and thrombocytopenia, documented by an erythrocyte count of 4.2 × 10^12^/L, hemoglobin level of 7.5 g/dL, and platelet count of 84 × 10^9^/L (see Figure 4). Clinically, this deterioration manifested as pronounced fatigue, reduced 6MWT performance, and an increase in NT-proBNP (see Figure 3), which was not interpreted as evidence of CTEPH progression in the absence of other findings suggesting pulmonary vascular deterioration. After careful evaluation, blood transfusion was considered necessary. The post-transfusion clinical response was delayed rather than immediate, with erythrocyte count increasing to 4.3 × 10^12^/L, hemoglobin to 8.0 g/dL, and platelet count to 94 × 10^9^/L. Minor gingival bleeding had been recorded before transfusion and may have contributed to the limited initial hematological and clinical response. Fatigue persisted for approximately two to three weeks before gradual improvement occurred. Importantly, no new major hemorrhagic events were reported after transfusion. At the latest follow-up, erythrocyte count had increased to 5 × 10^12^/L, hemoglobin to 9.0 g/dL, and platelet count to 105 × 10^9^/L.

## 4. Review and Discussion

### 4.1. Converging Prothrombotic Mechanisms Across Venous and Arterial Territories

This case illustrates the clinical relevance of several prothrombotic mechanisms converging in a single patient. The recurrent thrombotic phenotype cannot be explained by one isolated abnormality, but rather appears to reflect the cumulative and mutually reinforcing effects of myeloproliferative disease, inherited thrombophilic factors, cardiometabolic risk, and, later in the disease course, chronic hypoxemia secondary to progressive pulmonary vascular disease. This temporal evolution is important, as chronic hypoxia likely acted as an additional amplifier of an already established prothrombotic background rather than as the initial driver of disease. Such an evolving multifactorial profile is particularly difficult to manage in clinical practice, because each component may independently increase thrombotic risk while simultaneously reducing the therapeutic safety margin [4,9,13,32,33].

Polycythemia vera is a well-established risk factor for both arterial and venous thrombotic events, but the mechanisms responsible for this risk extend beyond a simple increase in erythrocyte count. Erythrocytosis and elevated hematocrit increase blood viscosity, reduce flow velocity, and favor vascular stasis, particularly in low-flow venous territories [4,13]. In addition, venous thrombi may become clinically relevant through embolic migration into the pulmonary arterial circulation, as illustrated by pulmonary embolism and, over time, chronic thromboembolic pulmonary hypertension [15,16]. However, the thrombotic phenotype of polycythemia vera is not limited to the venous system. Arterial involvement may occur through partially distinct mechanisms, including endothelial dysfunction, platelet activation, leukocyte activation, inflammatory signaling, high-shear vascular conditions, and interaction with traditional cardiovascular risk factors [6,34,35].

Qualitative abnormalities of red blood cells may further contribute to thrombogenesis, as erythrocytes in polycythemia vera may show increased adhesiveness to the endothelium and altered interactions with platelets and leukocytes. Activated platelets and leukocytes may amplify endothelial injury, release procoagulant mediators, and promote a more thrombogenic circulating environment. JAK2V617F-driven myeloproliferation appears to link clonal hematopoiesis with inflammation, endothelial dysfunction, and activation of cellular elements involved in coagulation [7,36].

In this patient, the prothrombotic background generated by polycythemia vera was further complicated by additional thrombophilic and cardiometabolic factors. Reduced protein C activity was particularly relevant, as it acts as a natural anticoagulant involved in the inactivation of factors Va and VIIIa [37,38]. Therefore, reduced activity may favor persistent thrombin generation and venous thrombus formation. Other detected variants may influence coagulation activation or fibrinolytic balance, although their independent clinical significance is variable. In particular, polymorphisms such as MTHFR C677T or PAI-1 4G/5G should not be overinterpreted in isolation. However, in a patient with recurrent thrombosis across multiple vascular territories, these abnormalities may contribute to the overall prothrombotic background when interpreted as part of a broader clinical phenotype rather than as isolated causal factors [9,11,12,39,40].

Obesity and arterial hypertension may have further amplified this thrombotic vulnerability from the early stages of disease. Obesity is associated with chronic low-grade inflammation, endothelial dysfunction, increased thrombin generation, impaired fibrinolysis, and altered adipokine-mediated vascular regulation. These mechanisms may interact with both inherited thrombophilia and polycythemia vera by promoting a proinflammatory and procoagulant vascular environment [41,42,43]. Arterial hypertension may add a further vascular component, particularly in arterial territories, by contributing to endothelial injury, vascular remodeling, and impaired vascular homeostasis [44,45]. Menopausal status and exposure to exogenous hormonal therapy should also be considered when evaluating thrombotic risk. Hormone replacement therapy or other estrogen-containing treatments may increase venous thromboembolic risk and may further amplify an already prothrombotic background [46,47]. Therefore, obesity, estrogen therapy and hypertension should not be considered primary explanations for the patient’s thrombotic phenotype, but rather additional factors that increased the susceptibility of an already prothrombotic system.

Later in the disease course, chronic hypoxemia secondary to progressive pulmonary vascular disease likely acted as an additional amplifying mechanism. Hypoxia may promote endothelial dysfunction, inflammatory activation, platelet reactivity, vasoconstriction, and impaired vascular reserve. In the setting of CTEPH, chronic hypoxemia may also contribute to systemic vulnerability by increasing cardiopulmonary stress and reducing tolerance to additional vascular events. In this patient, hypoxia therefore appears to represent a later superimposed factor that intensified an already established prothrombotic background rather than the initial driver of thrombosis [33,48,49,50].

However, in patients with recurrent or multisite thrombosis, especially when both venous and arterial territories are involved, the identification of one prothrombotic disorder should not preclude systematic evaluation for additional major thrombotic conditions. Malignancy, antiphospholipid syndrome, systemic vasculitis, paroxysmal nocturnal hemoglobinuria, inflammatory disorders, and other prothrombotic states may each contribute to thrombosis through distinct mechanisms [51,52].

Malignancy may promote thrombosis through tumor-related procoagulant activity, inflammation, endothelial activation, and treatment-related factors [53,54,55].

Antiphospholipid syndrome may cause both venous and arterial events through antibody-mediated endothelial, platelet, complement, and coagulation activation, and its diagnosis has direct therapeutic implications, particularly regarding the choice and duration of anticoagulation [56,57].

Systemic vasculitis may produce vascular occlusion through inflammatory vessel-wall injury, endothelial dysfunction, and secondary thrombosis, often requiring immunomodulatory therapy in addition to antithrombotic management [58,59].

Paroxysmal nocturnal hemoglobinuria may also be relevant in unexplained or unusual-site thrombosis, as complement-mediated hemolysis, nitric oxide depletion, platelet activation, and inflammatory mechanisms can generate a markedly prothrombotic state [60].

Therefore, in patients with complex thrombotic phenotypes, exclusion or identification of these additional conditions is essential, as it may substantially modify both etiological interpretation and long-term treatment strategy [9,56,59,60].

Taken together, these mechanisms support a cumulative and potentially synergistic model of thrombosis. Polycythemia vera provided the central myeloproliferative and inflammatory substrate, inherited thrombophilic factors impaired physiological anticoagulant and fibrinolytic balance, obesity and hypertension added cardiometabolic and endothelial stress and chronic hypoxemia later amplified systemic vascular vulnerability. This combination may explain why the patient’s thrombotic disease was recurrent, multisystemic, and involved both venous and arterial territories.

### 4.2. CTEPH: Disease Evolution and Management Constraints

Acute pulmonary embolism represents one of the most clinically important manifestations of venous thromboembolism because it can rapidly compromise pulmonary circulation and right ventricular function. In cases where the embolic burden is extensive, abrupt obstruction of the pulmonary arterial bed may increase pulmonary vascular resistance, produce acute right ventricular pressure overload, and lead to hemodynamic instability, syncope, or death [61,62]. In the presented case, the 2017 episode of massive pulmonary thromboembolism marked the first clearly documented pulmonary vascular event, with the association of severe dyspnea and presyncope reflecting the clinical severity of the acute episode.

However, the pulmonary history suggests that the 2017 event may not have represented the true beginning of the thromboembolic process. The patient had experienced recurrent dyspnea, dry cough, and asthenia since 2014, symptoms that were similar in character to those preceding the documented massive pulmonary embolism, although less severe and not investigated at that time. Furthermore, during the interval between the acute pulmonary embolism and the later diagnosis of CTEPH, the patient continued to report recurrent respiratory symptoms while receiving long-term anticoagulant therapy, without clear evidence of newly developed acute thromboembolic events on available imaging. One possible interpretation is that these episodes may have reflected recurrent small or clinically silent embolic events, progressive organization of previous thrombotic material, incomplete resolution of the initial thrombotic burden, or a combination of these mechanisms. This remains inferential, as no imaging confirmation was available for each symptomatic episode.

Although the patient’s PAWP of 14 mmHg remained within the diagnostic range for a precapillary hemodynamic profile, it was close to the upper limit. Therefore, the potential contribution of elevated left-sided filling pressures, volume status, and measurement conditions should be considered when interpreting the hemodynamic phenotype. Nevertheless, the markedly elevated pulmonary vascular resistance, persistent chronic thromboembolic obstruction on imaging, and the overall clinical context supported CTEPH as the predominant mechanism of a precapillary pulmonary hypertension.

CTEPH may develop through incomplete resolution of a major pulmonary embolic event, but it may also evolve through recurrent smaller pulmonary emboli that progressively obstruct and remodel the pulmonary vascular bed. In both scenarios, persistent organized thrombotic material may become incorporated into the pulmonary arterial wall, leading to fixed mechanical obstruction. Over time, secondary microvascular remodeling, endothelial dysfunction, inflammation, and increased pulmonary vascular resistance may develop, resulting in progressive pulmonary hypertension and right ventricular strain [15,16,17,63]. Therefore, the evolution from acute pulmonary embolism to CTEPH in this patient should be interpreted not as an isolated late complication, but as part of a prolonged pulmonary vascular disease trajectory occurring in the context of persistent symptomatic prothrombotic risk despite prescribed anticoagulation.

The management of CTEPH is distinct from that of other forms of pulmonary hypertension because potentially disease-modifying mechanical interventions are available.

Pulmonary endarterectomy remains the treatment of choice for patients with surgically accessible thromboembolic lesions and acceptable operative risk, as it can remove organized obstructive material from the pulmonary arteries and may offer definitive hemodynamic and clinical improvement [14,15,64].

Balloon pulmonary angioplasty has also become an important therapeutic option, particularly in patients with inoperable disease, distal lesions, or residual pulmonary hypertension after surgery [65,66].

However, both procedures require specialized expertise, careful anatomical selection, and experienced multidisciplinary CTEPH teams. Their implementation may be limited by local availability, patient comorbidities, procedural risk, or refusal of referral to an expert center, as occurred in the present case, in which pulmonary endarterectomy and balloon pulmonary angioplasty were not locally available, and the patient declined international transfer [67,68,69]. Consequently, targeted medical therapy with supplemental oxygen became the most feasible therapeutic approach.

Riociguat was selected as the initial CTEPH-directed vasodilator treatment because it is an approved soluble guanylate cyclase stimulator for symptomatic patients with inoperable CTEPH or persistent/recurrent pulmonary hypertension after pulmonary endarterectomy, with evidence supporting improvement in exercise capacity and pulmonary vascular resistance [70,71,72]. Locally, it also represented the only available dedicated pharmacological option for CTEPH. Despite riociguat therapy, the patient showed limited improvement, suggesting that monotherapy was insufficient to achieve stable disease control in the context of advanced CTEPH and substantial systemic comorbidity [73,74].

This limited response supported therapeutic escalation which was also consistent with the more recent approach in pulmonary hypertension management, which increasingly emphasizes early risk reassessment, treatment intensification, and combination therapy when monotherapy fails to achieve a low-risk profile [75,76,77,78,79,80].

Dual therapy was initiated once continuous subcutaneous treprostinil became available through the Romanian National Pulmonary Hypertension Program, where it was reimbursed according to the national regulatory framework for this indication. Its use was supported by the CTREPH trial, which demonstrated a significant improvement in exercise capacity, assessed by the 6-min walk distance after 24 weeks of high-dose subcutaneous treprostinil, in patients with severe inoperable CTEPH, as well as by current guideline recommendations allowing consideration of subcutaneous treprostinil in patients with inoperable CTEPH and WHO functional class III–IV [14,81,82]. Treprostinil is a prostacyclin analogue that promotes pulmonary and systemic vasodilation and may also exert antiplatelet, antiproliferative, and anti-inflammatory effects. Continuous subcutaneous administration is particularly relevant in severe pulmonary hypertension because it allows sustained drug delivery and persistent prostacyclin-pathway stimulation without the need for central venous access. However, this route also requires patient adherence, infusion-site care, dose titration, and monitoring for local or systemic adverse effects [82,83,84,85].

The initiation of dual pulmonary vasodilator therapy was followed by functional improvement and better oxygenation. However, later follow-up became more difficult to interpret after the development of significant anemia, as reduced exercise tolerance, fatigue, and biomarker deterioration such as increased NT-proBNP could no longer be attributed exclusively to the observed pulmonary vascular disease progression. Instead, these changes likely reflected the combined impact of cardiopulmonary limitation and hematological deterioration, as suggested by the parallel worsening of functional testing and hematological parameters shown in Figure 3 and Figure 4.

### 4.3. Cytoreduction and Thrombotic Risk Control

In this case, long-term anticoagulation was necessary but did not fully address the disease substrate maintaining thrombotic risk. The persistence of respiratory symptoms after the documented pulmonary embolic event, followed by later progression toward CTEPH, suggests that the prothrombotic state remained clinically relevant despite prescribed anticoagulation. Although recurrent silent pulmonary emboli cannot be proven without interval imaging confirmation, the disappearance of recurrent respiratory episodes after initiation of PV-directed therapy supports the clinical importance of controlling the underlying myeloproliferative process [86,87].

Therefore, cytoreductive therapy should be interpreted not only as hematological disease control, but also as a disease-directed component of thrombotic-risk reduction. In this patient, the therapeutic objective was not limited to normalization of blood counts, but also to reduction of the biological activity sustaining recurrent or progressive thrombotic manifestations [4,86].

Hydroxycarbamide was initially selected because it represents a standard first-line cytoreductive therapy in high-risk polycythemia vera, particularly in patients with previous thrombotic events or persistent elevation of hematological parameters. Its therapeutic rationale is based on reducing excessive myeloproliferation and improving control of erythrocyte, leukocyte, and platelet counts, thereby reducing hyperviscosity and cellular contributors to thrombosis [88,89]. In the present case, however, the response was not optimal, as the patient did not consistently achieve recommended therapeutic targets, particularly hematocrit control below 45% [34]. In this context, the switch to ruxolitinib was necessary.

Ruxolitinib is a JAK1/2 inhibitor that directly targets the dysregulated JAK–STAT signaling pathway central to JAK2V617F-positive polycythemia vera. Compared with nonspecific cytoreduction alone, ruxolitinib may provide more targeted control of the myeloproliferative process, with demonstrated benefit in hydroxycarbamide-resistant or intolerant PV, particularly regarding hematocrit control, splenomegaly reduction, and symptom improvement. In this patient, ruxolitinib therefore represented a more appropriate disease-directed option after insufficient hydroxycarbamide response, aiming to improve both hematological control and the systemic prothrombotic activity associated with active PV [90,91].

### 4.4. Neurological Implications

The neurological episode in this case was particularly significant because it involved concomitant arterial and venous cerebrovascular injury. Occlusion of the right vertebral artery at the V4 segment threatened posterior circulation inflow, while thrombosis of the right transverse–sigmoid sinus system impaired venous drainage. These two mechanisms have distinct but potentially convergent consequences: arterial occlusion may lead to brainstem, cerebellar, or posterior circulation ischemia, whereas cerebral venous sinus thrombosis may produce intracranial hypertension, cerebral edema, venous congestion and infarction, with or without hemorrhagic transformation and seizures. Their coexistence therefore created a high-risk neurological scenario, even in the absence of an initial parenchymal lesion [92,93,94,95].

The absence of arterial infarction, venous infarction, significant edema, or hemorrhagic transformation was most likely explained by preserved compensatory circulation at the time of presentation. On the arterial side, the anterior circulation remained patent, and the basilar artery was not involved, allowing continued perfusion of posterior circulation territories despite occlusion of the right vertebral artery at the V4 segment. In this setting, posterior circulation supply may have been maintained primarily through the contralateral vertebral artery and basilar artery, while additional collateral support through the circle of Willis, particularly via the posterior communicating arteries, may have helped preserve flow toward the posterior cerebral territories. This collateral reserve likely protected vulnerable brainstem, cerebellar, thalamic, and occipital regions from irreversible ischemic injury [96,97,98].

In parallel, the absence of major venous congestion-related parenchymal injury suggests that venous drainage was still partially compensated despite thrombosis of the right transverse–sigmoid sinus system. Drainage through the contralateral transverse and sigmoid sinuses, the torcular venous confluence, deep venous system, and cortical venous anastomoses may have limited venous pressure elevation and prevented progression toward venous infarction, cerebral edema, or hemorrhagic transformation. Therefore, the patient’s relatively limited neurological phenotype, consisting mainly of headache, central vertigo, balance impairment, and transient visual symptoms, likely reflected the preservation of both arterial inflow and venous outflow reserve at that moment, rather than the absence of significant cerebrovascular disease [22,99,100,101,102].

Pulmonary vasodilator therapy may also have contributed indirectly to neurological tolerance by improving oxygenation and reducing cardiopulmonary strain, thereby supporting systemic conditions necessary for cerebral perfusion [70,82,103]. In theory, improved oxygen delivery and more favorable hemodynamic status could help maintain cerebral vascular reserve in a patient with chronic hypoxemia and compromised posterior circulation [104]. However, a direct protective effect of riociguat or treprostinil on cerebral collateral perfusion cannot be demonstrated from the available data, and cerebral autoregulation remains influenced by multiple systemic and local factors.

The cognitive profile of such patients is also at risk. Chronic hypoxemia may impair cerebral metabolism and oxygen-dependent neuronal function, particularly in domains such as attention, processing speed, executive function, and memory [19,105]. In parallel, persistent vascular injury, reduced cerebrovascular reserve, systemic inflammation, anemia, and polycythemia-related microcirculatory impairment may further reduce cerebral tolerance to metabolic or hemodynamic stress [103,106,107,108]. In this context, the borderline MoCA result and mild deficits in memory and attention are clinically relevant, suggesting early cognitive impairment rather than isolated test variability. Therefore, the patient’s mild cognitive impairment most likely reflects the combined impact of chronic hypoxia, systemic prothrombotic disease, and persistent cerebrovascular pathology [109,110].

Overall, the patient’s neurological status remains highly vulnerable despite clinical improvement. Chronic vertebral artery occlusion and persistent transverse–sigmoid sinus thrombosis leave little margin for additional arterial or venous cerebral events, while the observed hemorrhagic tendency makes overtreatment equally dangerous. This dual risk transforms subsequent medication management into a narrow balance between preventing further thrombosis and avoiding potentially catastrophic intracranial bleeding [111,112,113].

### 4.5. Therapeutic Options Under Competing Thrombotic and Hemorrhagic Risks

The main therapeutic challenge in this case was not the isolated selection of individual drugs, but the cumulative effect of several necessary treatments acting within a narrow safety margin. Apixaban, treprostinil, and ruxolitinib each addressed a distinct and essential component of the patient’s disease: prevention of recurrent thrombosis, stabilization of severe CTEPH, and control of the polycythemia vera-related myeloproliferative substrate. However, their combined administration and long-term adjustment proved highly challenging, requiring continuous multidisciplinary reassessment in order to maintain the delicate balance between thrombotic recurrence and hemorrhagic complications [114,115].

Apixaban is a direct oral anticoagulant that selectively inhibits activated factor X, thereby reducing thrombin generation and fibrin clot formation. After oral administration, it reaches peak plasma concentration within approximately 3–4 h and has an apparent half-life of about 12 h, allowing sustained anticoagulant activity with repeated dosing. It is eliminated through multiple pathways, including hepatic metabolism, biliary and direct intestinal excretion, and renal clearance, with renal elimination accounting for approximately one quarter of total clearance. It is also a substrate of CYP3A4 and P-glycoprotein, making its exposure potentially sensitive to strong inhibitors or inducers of these pathways. Its main adverse effect is bleeding, ranging from mucosal or gastrointestinal bleeding to potentially severe hemorrhagic complications, including intracranial hemorrhage [116,117].

Ruxolitinib is an oral Janus kinase 1 and 2 inhibitor used as disease-directed therapy in polycythemia vera with inadequate response or intolerance to hydroxycarbamide. By inhibiting JAK1/2 signaling, it reduces dysregulated JAK–STAT pathway activation, which is central to JAK2V617F-positive myeloproliferation. After oral administration, ruxolitinib is rapidly absorbed, with peak plasma concentrations reached within approximately 1–2 h, and it has a relatively short elimination half-life of approximately 3 h. It is metabolized mainly in the liver, predominantly through CYP3A4, and eliminated largely as metabolites through urine and, to a lesser extent, feces. Its main adverse effects are hematological, particularly anemia, thrombocytopenia, and neutropenia, reflecting the role of JAK2 signaling in normal hematopoiesis. Infectious complications may also occur and require clinical monitoring [91,118].

Treprostinil is a prostacyclin analogue used as pulmonary vasodilator therapy in severe pulmonary hypertension, including selected cases of non-operable CTEPH. It acts through the prostacyclin pathway, promoting pulmonary and systemic vasodilation and inhibiting platelet aggregation, with additional potential antiproliferative and anti-inflammatory effects in the pulmonary vasculature. In this patient, treprostinil was administered as a continuous subcutaneous infusion, a route that allows sustained drug delivery and persistent prostacyclin-pathway stimulation without the need for central venous access. Its terminal half-life after subcutaneous administration is approximately 4 h. Treprostinil is metabolized mainly in the liver, primarily through CYP2C8, and is eliminated predominantly as metabolites in urine and feces. Its main adverse effects include infusion-site pain or reactions, headache, flushing, diarrhea, nausea, jaw pain, hypotension, and increased bleeding tendency related to inhibition of platelet aggregation [83,85,119].

When considered together, these therapies did not suggest a single dominant pharmacokinetic interaction explaining the patient’s bleeding risk. Apixaban and ruxolitinib are both influenced by CYP3A4-mediated metabolism, but neither drug is primarily used as a strong CYP3A4 inhibitor or inducer of the other. In addition, available pharmacokinetic data do not suggest that ruxolitinib has a dominant clinically relevant P-glycoprotein-mediated transporter effect that would be expected to substantially increase apixaban exposure. Treprostinil is metabolized mainly through CYP2C8 rather than CYP3A4. Therefore, the main therapeutic concern was not a direct metabolic or transporter-mediated interaction between the three agents, but a cumulative pharmacodynamic effect on hemostasis and hematological tolerance.

The cumulative hemorrhagic risk was clinically relevant because each therapy affected hemostasis or hematological tolerance through a different mechanism. Apixaban provided necessary anticoagulation through factor Xa inhibition. Ruxolitinib may have contributed to anemia and thrombocytopenia through myelosuppressive effects, while treprostinil may have further increased bleeding tendency through inhibition of platelet aggregation. Although the antiplatelet effect of treprostinil is pharmacologically recognized, the magnitude of its additional contribution to bleeding risk in this specific therapeutic context remains insufficiently defined in the available literature. Nevertheless, given the coexistence of anticoagulation, cytopenias, and prostanoid-related platelet inhibition, a cumulative contribution to hemorrhagic vulnerability was clinically plausible. This was reflected by macroscopic hematuria and gingival bleeding in the presented case, while also raising concern for more severe potential complications, including gastrointestinal bleeding or intracranial hemorrhage.

Despite this hemorrhagic risk, complete interruption of anticoagulation represented the least acceptable option, as its discontinuation was followed by severe neurological thrombotic complications. Hepatic and renal function remained preserved throughout the observation period and as such, clinically relevant accumulation of apixaban due to renal impairment was not considered a contributor to the patient’s bleeding risk. Therefore, management of bleeding risk had to focus primarily on correcting the modifiable contributors to cytopenia and impaired hemostasis while preserving antithrombotic protection.

In practical terms, management required close hematological monitoring and repeated reassessment of the concomitant therapies. Ruxolitinib dosing was adjusted according to hematological tolerance, with previous optimization up to 15 mg twice a day and subsequent reduction to 5 mg twice a day when cytopenias became clinically significant. Mild-to-moderate anemia was initially tolerated under close surveillance, but significant cytopenias prompted consideration of dose reduction or temporary treatment interruption. Treprostinil dosing was guided primarily by pulmonary vascular response, functional status, and tolerability, but was also reassessed in the setting of thrombocytopenia and clinically relevant bleeding episodes. Apixaban was maintained at 5 mg twice daily because of the patient’s very high thrombotic risk, with dose appropriateness repeatedly reviewed, while supportive measures, including transfusion, were used when anemia became symptomatic. Although no universal platelet threshold can fully determine anticoagulation safety in such a complex case, the patient’s platelet counts were interpreted in relation to commonly used clinical thresholds for therapeutic anticoagulation in thrombocytopenic patients, with counts above approximately 50 × 10^9^/L generally supporting continuation when thrombotic risk is high and active major bleeding is absent [120].

This approach reflects the central therapeutic dilemma of the case: anticoagulation remained mandatory, but the surrounding therapies required continuous adjustment to prevent thrombocytopenia, anemia, and bleeding from becoming life-threatening.

## 5. Limitations

This study has several limitations related to its design. First, it is based on a single complex case involving several rare and overlapping pathologies, which limits the possibility of drawing broad conclusions regarding causality, prognosis, or optimal therapeutic strategy. Second, because the full association of polycythemia vera, inherited thrombophilia, CTEPH, chronic hypoxia, cerebral venous sinus thrombosis, and vertebral artery occlusion appears to be extremely uncommon, the available literature does not provide sufficient comparative data or standardized management pathways for this exact clinical scenario. Therefore, the review component of this article necessarily follows a narrative and integrative approach rather than a systematic or meta-analytic design. Finally, therapeutic interpretation was based on real-world longitudinal management and multidisciplinary clinical reasoning, which reflects practical complexity but cannot establish definitive treatment superiority between different possible strategies.

## 6. Conclusions

Improved survival in patients with severe multisystemic conditions may increase the clinical relevance of complex presentations requiring coordinated management. This case illustrates how overlapping rare pathologies and multiple essential therapies can create therapeutic conflicts that are difficult to solve within existing evidence-based frameworks. Further literature is needed on highly complex patients requiring simultaneous therapies, with particular focus on safer targeted strategies that reduce cumulative adverse effects while preserving disease control.

## Data Availability

The data is not publicly available. Anonymized data may be provided upon request from the principal authors.
